# Postoperative respiratory adverse events in pediatric anesthesia: Risk factors, clinical implications, and management strategies

**DOI:** 10.1097/MD.0000000000044598

**Published:** 2025-12-26

**Authors:** Fang Li, Xiaoyan Zhou, Lihua Jiang, Yuhua Wu, Anping Zhang, Huan Yang, Benzhen Chen

**Affiliations:** a Department of Anesthesia and Surgery Center, Sichuan Provincial Women’s and Children’s Hospital, the Affiliated Women’s and Children’s Hospital of Chengdu Medical College, Chengdu, Sichuan, China; b Department of Operating Room Nursing, Sichuan Provincial People’s Hospital, University of Electronic Science and Technology of China, Chengdu, People’s Republic of China.

**Keywords:** anesthetic management, clinical outcomes, pediatric anesthesia, postoperative respiratory adverse events, risk factors

## Abstract

This review aimed to comprehensively assess the risk factors, clinical implications, and management strategies related to postoperative respiratory adverse events (RAEs) in pediatric anesthesia, particularly focusing on children recovering from general anesthesia. An extensive literature search was conducted across PubMed, Embase, and the Cochrane Library, prioritizing studies published from 2000 to 2025 that addressed RAEs in pediatric populations. The selected studies included randomized controlled trials, observational studies, and systematic reviews, focusing on various aspects, such as the identification of risk factors, the physiological vulnerabilities of children, and effective clinical interventions. A categorized analytical framework was employed to synthesize the findings related to risk factors, RAEs outcomes, and management strategies. The review identified multiple intrinsic and extrinsic risk factors for RAEs in pediatric patients, notably age (especially neonates and infants), recent upper respiratory infections, preexisting respiratory conditions, and specific procedural factors, such as the choice of anesthesia induction method (intravenous vs inhalational). Significant adverse outcomes, such as laryngospasm, bronchospasm, apnea, and pneumonia, often stem from the unique anatomical and physiological features of children, as well as inadequacies in postoperative management practices. Enhanced Post-Anesthesia Care Unit (PACU) monitoring and personalized anesthetic regimens have emerged as critical strategies to mitigate these risks. Furthermore, this review emphasizes the role of biomarkers, such as red cell distribution width, in predicting postoperative respirator complications. These findings suggest that a multifaceted approach is essential to reduce the incidence and severity of RAEs in pediatric anesthesia. Emphasizing the early identification of at-risk patients, tailoring anesthetic plans, and structured postoperative monitoring can significantly enhance clinical outcomes and resource optimization within healthcare systems. Continued research on predictive modeling and collaborative studies is paramount for developing evidence-based strategies that address this critical aspect of pediatric anesthesia care.

## 1. Introduction

### 1.1. Background and significance

#### 1.1.1. Rising pediatric surgical demands and the prevalence of RAEs

Over the past decades, pediatric surgical demands have surged globally, a trend driven by advancements in surgical techniques, anesthetic management, and improved survival rates in children with complex congenital diseases^.[1]^ This escalation in pediatric procedures has been accompanied by an increased risk of complications during the postoperative recovery period, among which RAEs are a frequent and critical concern. RAEs encompass a range of conditions such as laryngospasm, bronchospasm, decreased oxygen saturation, apnea, and pneumonia, which can complicate the recovery process following general anesthesia.^[1]^

Available data indicate that RAEs are disproportionately prevalent in pediatric populations compared with adults because of their unique anatomical and physiological features. Furthermore, children undergoing procedures under general anesthesia are more susceptible to airway complications, accounting for significant morbidity. For instance, studies have demonstrated an increased frequency of airway interventions among pediatric patients presenting with preexisting conditions, such as upper respiratory infections (URIs).^[1]^ The significant prevalence and impact of RAEs underscore the importance of identifying and mitigating their root causes to ensure safer surgical outcomes in this vulnerable population.

#### 1.1.2. Anatomical and physiological susceptibilities in children during the recovery from general anesthesia

Children possess distinct anatomical and physiological features that increase their vulnerability to RAEs during postoperative recovery from general anesthesia. Notably, pediatric airways are narrower, softer, and more easily collapsible than those of adults, attributes that predispose children to airway complications, such as obstruction or bronchospasm.^[1]^Additionally, reduced lung capacity and immature respiratory control mechanisms impair the effective clearance of secretions and exacerbate the risk of potentially fatal events, such as apnea or hypoxia.^[1]^

Specific predispositions such as weaker pharyngeal musculature and higher airway resistance further present challenges in ensuring proper ventilation during recovery from general anesthesia.^[1]^ These vulnerabilities are particularly critical for neonates and infants, whose fragmented sleep cycles and immature immune systems may increase the likelihood of adverse airway events during postoperative care. Thus, a comprehensive understanding of pediatric anatomical and physiological susceptibilities is essential to optimizing procedural planning and effectively mitigating RAEs.

#### 1.1.3. Clinical consequences and resource implications of RAEs

RAEs in pediatric patients have significant implications for clinical management and resource allocation, as these complications can lead to prolonged hospital stays, repeated interventions, and increased healthcare costs. From a clinical standpoint, RAEs during the postoperative recovery period may delay the discharge process or require higher levels of care, such as intensive monitoring or supplemental oxygenation.^[1]^ Moreover, conditions such as aspiration pneumonia or prolonged apnea can result in life-threatening consequences, increasing patient mortality risk and burdening health providers with difficult ethical and logistical decisions.^[1]^

The economic impact of RAEs is equally concerning, as recurrent complications demand additional utilization of medical resources, including the extended use of Post-Anesthesia Care Unit (PACU) and respiratory support technology.^[1]^ Such requirements can stretch the capacities of healthcare systems, especially in resource-limited settings or during periods of heightened surgical demand. Identifying the key risk factors contributing to RAEs offers a pathway to not only enhance the quality of perioperative care but also mitigate the broader resource challenges faced by pediatric hospitals and surgical centers.

### 1.2. Focus and scope

#### 1.2.1. Target population: children recovering from general anesthesia

This narrative review specifically focuses on children ranging from neonatal to school age, who are undergoing the recovery phase following general anesthesia. Such children face inherent physiological and anatomical challenges that make them prone to adverse respiratory events.^[1]^ By narrowing the target population to this demographic, this review aims to elucidate specific vulnerabilities, examine strategies for risk minimization and improve postoperative management.

#### 1.2.2. High-risk groups: age groups, comorbidities, and preexisting respiratory issues

The variability in risks related to RAEs necessitates the identification and evaluation of high-risk groups within the pediatric population. Evidence suggests that neonates, infants, and children with preexisting respiratory conditions, such as asthma or sleep-disordered breathing, are particularly susceptible to postoperative respiratory complications.^[1]^ In addition, recent or ongoing URIs significantly increase the risk of adverse airway events during recovery. For instance, a study analyzing pediatric patients undergoing procedural sedation confirmed the association between URIs status and an increased incidence of airway interventions and RAEs.^[1]^

These findings emphasize the need to adopt targeted approaches for managing children presenting with comorbidities and underlying respiratory issues before general anesthesia is administered. Focused attention on high-risk subgroups can facilitate individualized care and outcome optimization.^[1]^

### 1.3. Objectives and clinical relevance

#### 1.3.1. Identification and categorization of risk factors for RAEs

The primary objective of this review was to comprehensively identify and categorize the risk factors contributing to RAEs in children recovering from general anesthesia. As research has already established correlations between factors such as URIs status and adverse airway events, this review aims to extend this knowledge by synthesizing findings across multiple studies.^[1]^ By highlighting procedural, patient-related, and management-associated risks, evidence-based insights conducive to mitigating the prevalence and impact of RAEs will be provided.

#### 1.3.2. Implications for clinical optimization and practice improvement

Understanding the factors underlying RAEs has profound implications in clinical anesthetic practice. Early identification and stratification of high-risk patients offer an opportunity to tailor preoperative preparation and intraoperative anesthetic management.^[1]^ Furthermore, such insights can underlie enhanced postoperative monitoring protocols, ensuring earlier detection and intervention of respiratory complications. This review not only underscores the value of optimizing perioperative care strategies through risk mitigation but also prompts a larger conversation about resource allocation in pediatric healthcare systems facing growing surgical demands.

## 2. Methodology

### 2.1. Literature search strategy

#### 2.1.1. Databases utilized: PubMed, Embase, Cochrane Library

The study was conducted at Sichuan Provincial Women’s and Children’s Hospital. The Medical Ethics Committee of Sichuan Provincial Women’s and Children’s Hospital approved the present study (review board number: 20241110-111). The search for relevant literature commenced with 3 principal databases: PubMed, Embase, and Cochrane Library. PubMed was prioritized for its extensive catalog of biomedical literature, particularly in pediatric anesthesia and the study of postoperative RAEs. This database, which is the gold standard for indexing peer-reviewed articles from various medical journals, is essential for identifying studies that discuss physiological and clinical factors contributing to RAEs in children. EMBASE complements PubMed with a broader scope, including a substantial number of conference abstracts and European journals, which are pertinent for identifying additional regional studies and systematic reviews. Finally, the Cochrane Library specializes in systematic reviews and meta-analyses, ensuring that the review incorporates evidence-based interventions and key methodological findings related to the prevention and management of RAEs during postoperative recovery in pediatric patients. Using these databases has enabled a comprehensive and multidimensional exploration of the topic, covering clinical studies as well as expert consensus guidelines.^[2]^

#### 2.1.2. Inclusion and exclusion criteria for study selection

Inclusion criteria were established to ensure the relevance and quality of the analyzed studies. Eligible studies were those with a focus on pediatric populations aged 0 to 18 years recovering from general anesthesia; a quantitative or qualitative assessment of RAEs during the postoperative period; study designs including randomized controlled trials (RCTs), observational (prospective or retrospective cohort) studies, and systematic reviews; and publication in peer-reviewed journals indexed within PubMed, Embase, and the Cochrane Library. Additionally, studies that explored the risk factors, physiological vulnerabilities, and impact of anesthesia techniques in pediatric patients have been emphasized.

The exclusion criteria were studies centered on adult populations, those examining unrelated postoperative complications, and publications lacking an explicit focus on respiratory outcomes. Articles discussing experimental animal models, non-English language studies, or anecdotal reports were excluded to maintain the scientific rigor of the narrative review. Furthermore, studies older than 20 years were generally excluded, except for seminal works referenced in newer research on historical contexts and analysis.^[2]^

#### 2.1.3. Keywords and search periods used

The search strategy employed specific keywords connected to the scope of RAEs and pediatric anesthesia, including combinations such as “pediatric anesthesia,” “postoperative respiratory adverse events,” “perioperative complications,” “laryngospasm,” “bronchospasm,” “hypoxia,” and “pulmonary function tests.” Boolean operators were strategically applied to refine search results, crafting search strings like (“pediatric anesthesia” AND “respiratory complications”) OR (“postoperative” AND “respiratory adverse events”).

The search was time-bound to include studies published between 2000 and 2024, with a focus on recent literature that reflects advancements in pediatric anesthesia practices, tools, and management strategies. The selected timeline aligns with the availability of comprehensive clinical guidelines in this field, such as the 2024 consensus report on pediatric perioperative risk prediction.^[2]^ This period also captures technological advancements and a growing emphasis on targeted anesthetic management in children during the postoperative recovery phase.

### 2.2. Study types and data extraction

#### 2.2.1. Types of studies included (e.g., RCTs, prospective and retrospective cohort studies)

The narrative review prioritized various study designs that contributed high-quality evidence to the research question. RCTs were included owing to their robust methodology and ability to identify causative relationships, particularly in interventions targeted at minimizing RAEs. Prospective and retrospective cohort studies are essential for analyzing broader patient populations over defined timeframes, providing critical insights into risk factors and trends specific to pediatric responses to general anesthesia, as suggested in the clinical consensus guidelines.^[2]^ Observational studies have played a supporting role in identifying correlations between preoperative conditions and adverse respiratory events.

Systematic reviews and meta-analyses were also incorporated to leverage existing pooled data and expert evaluations across multi-institutional studies, ensuring a reliable and overarching understanding of RAEs. The choice of study type underscores the effort to utilize diverse methodological strengths to build a comprehensive narrative framework.

#### 2.2.2. Methodological criteria for quality assessment

Stringent evaluation criteria were applied to assess study quality to ensure the reliability and validity of the conclusions drawn,. The CONSORT guidelines for RCTs were followed to evaluate the adequacy of their design, implementation, and reporting. Observational studies were reviewed using the STROBE checklist, which assesses study design integrity, population selection, and statistical analysis reliability.

For systematic reviews, adherence to the PRISMA statement assured transparent reporting, comprehensive search strategies, and critical evaluation of primary studies. This quality assessment process was further informed by expert recommendations for perioperative risk prediction in the pediatric population.^[2]^ The application of these standardized tools facilitated the identification of potential biases and ensured that the analyzed data were representative and methodologically sound.

### 2.3. Analytical framework

#### 2.3.1. Categorization of data based on risk factors, adverse event outcomes, and interventions

The data extracted from the reviewed studies were systematically categorized into 3 primary dimensions: risk factors, adverse event outcomes, and clinical interventions. The risk factors analyzed included patient-related variables such as age subsets (neonates, infants, and older children), comorbid conditions (e.g., asthma, and sleep-disordered breathing), and recent URIs.^[2,3]^ Adverse event classifications encompassed typical RAEs such as laryngospasm, bronchospasm, and postoperative hypoxia.

Interventions reflect both preventive and reactive strategies employed during pediatric anesthesia, such as the utilization of cuffed endotracheal tubes (ETTs) and tailored anesthetic regimens.^[2]^ The categorized data were synthesized into actionable insights to illuminate patterns and relationships that informed clinical decision-making, emphasizing areas requiring vigilance and optimization in anesthesia practices.

This categorization framework allowed not only a comprehensive overview of recorded RAEs but also for an evaluation of intervention efficacy. The findings were systematically interpreted to align with recommendations in expert consensus documents regarding pediatric perioperative assessment and risk management.^[2]^ Following this approach, we ensured that the narrative review upheld scientific rigor while distilling the findings into practical implications.

## 3. RAEs: definitions and clinical characteristics

### 3.1. Typology of RAEs

#### 3.1.1. Laryngospasm and bronchospasm

Laryngospasm and bronchospasm are among the most critical RAEs encountered during recovery from pediatric anesthesia. Laryngospasm refers to prolonged involuntary contraction of the vocal cords resulting in airway obstruction and can lead to hypoxia, bradycardia, or cardiac arrest if severe or persistently untreated. Bronchospasm, in contrast, is a sudden narrowing of the bronchioles due to tightening of the airway smooth muscles, increased mucus production, and inflammation. Both are more common in children than in adults because of their smaller and more reactive airways compared to adults.^[4,5]^

In a multicenter observational study across Europe, difficulties in airway management were identified as a significant contributor to critical respiratory events, with repeated attempts to secure the airway increasing the likelihood of complications, such as laryngospasm and bronchospasm.^[4]^ Additionally, African American children are reported to have a higher incidence of respiratory events, with bronchospasm being particularly prevalent in this demographic.^[6]^ RAEs are frequently associated with otolaryngology procedures.^[6]^ These findings underscore the importance of tailored approaches for monitoring and managing airway responses during postoperative recovery.

#### 3.1.2. Decreased oxygen saturation and respiratory obstruction

Decreased oxygen saturation, defined as a drop in SpO_2_ to dangerous levels, frequently occurs following pediatric anesthesia due to airway obstruction or inadequate ventilation. Obstructions may arise from anatomical predispositions such as enlarged tonsils or adenoids, increased airway reactivity, or equipment-related issues, such as improper airway device positioning. If not identified and managed promptly, decreased oxygen saturation can lead to hypoxemia, prolonged recovery, or reintubation.^[4,5]^

A study involving neonates recovering from anesthesia revealed a significant association between low postmensural ages and major respiratory events, such as upper airway obstruction and subsequent low oxygen saturation.^[7]^ The timing of extubation was also identified as a critical factor, with improper timing increasing the risk of postoperative desaturation. For instance, extubation during pediatric anesthesia may precipitate coughing, airway irritation, or complete airway collapse.^[5]^

#### 3.1.3. Postoperative apnea and pneumonia

Postoperative apnea is a common concern, especially in premature neonates or infants, owing to their immature central respiratory control and increased sensitivity to anesthetic agents. Apnea episodes can result in significant hypoxemia and bradycardia and, in severe cases, necessitate reintubation and prolonged ventilatory support.^[7]^

Postoperative pneumonia, though less frequent, remains a severe RAEs linked to prolonged intubation or inadequate airway clearance. For children undergoing procedures such as thoracic surgery, there is a risk of bacterial colonization of the lungs, which may lead to pneumonia and extended hospital stays.^[4,8]^ Furthermore, this complication is exacerbated in children with underlying conditions such as obstructive sleep apnea or immunosuppressive states.^[9]^

### 3.2. Age-related physiological factors

#### 3.2.1. Risk variations between neonates, infants, and older children

Respiratory risks following anesthesia recovery vary significantly across pediatric age groups owing to physiological and anatomical differences. Neonates and infants have underdeveloped lungs and narrow airways, making them more prone to obstruction. Their respiratory control centers are immature, increasing the likelihood of apnea or hypoventilation compared to older children.^[5,7]^

In contrast, older children tend to have more robust airway anatomy but may still develop RAEs due to particular risk factors such as obesity or comorbidities such as asthma. For instance, obese children demonstrate a higher incidence of perioperative RAEs during ambulatory anesthesia due to compromised lung mechanics and airway reactivity.^[10]^

#### 3.2.2. Structural predispositions impacting airway management

Structural factors such as enlarged tonsils, adenoids, and congenital airway abnormalities significantly influence airway management in pediatric patients. In children undergoing adenotonsillectomy, the presence of obstructive sleep apnea further complicates perioperative respiratory outcomes. Structural immaturity in neonates, including floppy airways and underdeveloped cartilage, creates challenges during intubation and extubation.^[4,5]^

In a European multicenter study, challenging airway management due to anatomical predispositions was closely related to increased respiratory complications such as asthma/wheezing/snoring/passive smoking.^[4]^ The study advocates heightened stratification during preoperative assessment to effectively address these inherent airway difficulties.

### 3.3. Association with surgical types

#### 3.3.1. Adenotonsillectomy and other ENT surgeries

Adenotonsillectomy is a common procedure linked to high rates of RAEs due to preexisting conditions like obstructive sleep apnea and airway hyperresponsiveness. Respiratory complications such as laryngospasm, decreased oxygen saturation, and postoperative apnea are frequent in children with severe obstructive sleep apnea undergoing adenotonsillectomy, a retrospective study of children admitted to the pediatric intensive care unit (PICU) after adenotonsil-lectomy between 08/2006 and 09/2015. Demographics, risk factors, and occurrence of adverse postoperative respiratory events (AEs) (oxygen saturation < 92, stridor, bronchospasm, pneumonia, pulmonary edema, re-intubation) were recorded. During the studied time period 4029 tonsil/adenoid procedures were performed in 3997 children. 179, admitted to the PICU postoperatively, met criteria for analysis. PICU adverse AEs occurred in 59%:44–83% in any particular risk category. PACU adverse AEs occurred in 42%. Of those with PACU events: 92% suffered AEs in the PICU; however, 35% of those without a PACU AEs still suffered a PICU AEs.^[9,11]^

Empirical evidence further highlights the risks among high-risk pediatric patients, with studies indicating that PACU complications do not reliably predict later adverse events in the PICU.^[9]^ The role of risk stratification in identifying and managing these complications has been emphasized in recent research.^[11]^

#### 3.3.2. Procedures performed under ambulatory anesthesia

Children undergoing ambulatory anesthesia, including those with postoperative desaturation and obstruction, are at particular risk for RAEs. Ambulatory settings, while convenient, often lack immediate access to critical care support, preemptively increasing the stakes to identify and mitigate risk factors preemptively.^[10]^

Children with underlying obesity develop RAEs more frequently during ambulatory anesthesia because of exacerbated airway obstruction and compromised pulmonary function.^[10,11]^ Optimizing drug dosing and ensuring continuous monitoring are the recommended strategies to mitigate these risks.

#### 3.3.3. Gastrointestinal and thoracic surgeries

Gastrointestinal and thoracic surgical procedures pose unique respiratory challenges during recovery from anesthesia. Thoracic surgeries, in particular, may exacerbate airway inflammation and lung collapse, contributing to postoperative pneumonia and other respiratory complications.^[8,12]^

Patients undergoing gastrointestinal surgeries may also develop RAEs such as decreased oxygen saturation or obstructive apnea owing to postoperative positioning and intraoperative airway manipulations.^[12]^ Thus, careful surgical planning and proper patient positioning after surgery are critical for preventing respiratory complications.

## 4. Analysis of risk factors

### 4.1. Patient-related risk factors

#### 4.1.1. Age and weight thresholds impacting RAEs

Age and weight thresholds are critical intrinsic factors influencing the risk of RAEs during pediatric postoperative anesthesia recovery. Neonates and infants, especially those younger than 1 year, are highly vulnerable to RAEs due to immature physiological structures and limited compensatory capacity for hypoxemia and airway compromise. Robert study has shown that children younger than 1 year and those below 5 years of age have an elevated risk of adverse respiratory outcomes, specifically during the postoperative emergence and recovery phases, they found that in 26 cases of anesthesia-related pediatric cardiac arrests, 67% in children.^[13,14]^ For neonates requiring surgery, lower birth weight (<1.58 kg) and a postmenstrual age below 41 weeks are independently associated with a significantly increased risk of major respiratory events.^[7]^ The interaction between weight thresholds and anesthesia recovery also correlates with physiological fatigue and airway complexity, particularly in younger pediatric populations.

Overweight and obese children face distinct challenges during recovery from anesthesia. Obesity is associated with perioperative RAEs due to difficulty in airway management, reduced pulmonary compliance, and an increased risk of hypoventilation.^[10]^ Similarly, underweight children (<18kg) undergoing adenotonsillectomy face heightened risks of postoperative respiratory complications, emphasizing the need for vigilant postoperative observation.^[15]^ These findings underscore the importance of adapting anesthesia strategies based on weight and age to adequately mitigate RAEs in diverse pediatric population subsets.

#### 4.1.2. Comorbidities: sleep-disordered breathing, asthma, congenital abnormalities

Comorbid conditions considerably enhance RAEs risks in pediatric patients undergoing general anesthesia. Sleep-disordered breathing, including obstructive sleep apnea, is a well-documented risk factor for postoperative respiratory complications. Children with preoperative polysomnography-confirmed Sleep-disordered breathing demonstrate significantly higher incidences of postoperative RAEs, driven by hypoxia and mechanical airway obstruction.^[16]^ Elevated red cell distribution width values, associated with hypoxia-mediated inflammation, have been identified as predictors of RAEs in children undergoing adenotonsillectomy for sleep-disordered breathing.^[17]^

Asthma is another critical factor associated with perioperative respiratory events that contributes to bronchospasm and exacerbation during recovery. Preoperative characterization and stabilization of asthmatic children significantly reduce the incidences of postoperative respiratory events.^[6]^ Congenital abnormalities, including craniofacial dysmorphology and respiratory muscle weakness observed in syndromes such as cardiofaciocutaneous syndrome, further increase postoperative risks, necessitating specialized airway management techniques.^[18]^ These comorbidities illustrate the importance of preoperative consultations, customized anesthesia regimens, and postoperative surveillance to reduce RAEs in vulnerable pediatric populations.

#### 4.1.3. Recent URIs and airway hypersensitivity

URIs remain a definitive risk factor for perioperative and postoperative respiratory complications. Evidence indicates a statistically significant increase in airway adverse events in pediatric patients with active or recent URIs conditions,^[1]^ with the highest risk observed in patients presenting with thick secretions within the airway during infection.^[19]^ As symptomatic URIs compromise mucociliary clearance, airway reactivity and hypersensitivity following anesthesia are heightened, leading to an increased likelihood of laryngospasm, bronchospasm, and hypoxemia during recovery.^[20]^ For children experiencing URIs symptoms, a symptom-free interval of 1-2 weeks before surgery substantially reduces RAEs.^[19]^ The ability to predict RAEs through systematic URIs assessment allows for informed scheduling changes and mitigation during postoperative care planning.^[20]^

### 4.2. Procedure and anesthetic correlates

#### 4.2.1. Induction technique: intravenous versus inhalational anesthesia

Anesthetic induction techniques are significant determinants of the incidence of respiratory complications, and comparative studies have demonstrated that intravenous propofol induction markedly reduces RAEs relative to inhalational sevoflurane induction. In high-risk pediatric populations, RAEs are observed less frequently during intravenous induction (26% vs. 43%) particularly during induction itself (11% vs. 32%).^[21]^ Propofol’s favorable pharmacokinetics—rapid elimination and lower airway irritation—make it particularly advantageous in children predisposed to hypersensitive airway responses.^[22]^ Conversely, inhalational techniques may trigger bronchospasm and laryngospasm, increasing the risk of airway obstructions during emergence from anesthesia.^[22]^ Clinicians should weigh risk factors such as anxiety, age, and preexisting respiratory conditions when customizing induction methodologies.

#### 4.2.2. Use of cuffed versus uncuffed ETTs in patients with hypersensitivity

The choice between cuffed and uncuffed ETTs plays a critical role in mitigating RAEs, particularly in pediatric patients with airway hypersensitivity. Cuffed ETTs provide effective airway sealing, reduce aspiration risks and facilitate controlled ventilation. However, they may exacerbate airway reactivity, particularly in children with significant mucus secretion or airway hypersensitivity. Conversely, while uncuffed ETTs minimize tracheal mucosal irritation, they risk inadequate ventilation in conditions requiring precise pressure adjustments.^[4]^ The APRICOT study emphasized the importance of stratifying ETTs choices based on individual risk profiles, suggesting that cuffed ETTs are reserved for patients requiring enhanced ventilatory control.^[4]^ Balancing airway safety is pivotal for preventing critical respiratory outcomes.

#### 4.2.3. Anesthesia duration and type of surgery

Prolonged anesthesia duration correlates significantly with increased respiratory complications and hospital admissions in pediatric postoperative recovery. Patients requiring extended anesthesia exhibit heightened physiological fatigue, predisposing them to hypoventilation and airway collapse, in Landry Elizabeth article, their multivariate analysis found that compared with shorter cases, cases lasting between 61 and 180 minutes were associated with a higher risk of unplanned postoperative ICU admissions (OR: 3.73, 95% CI: 2.19–6.36).^[14,15]^ Surgical types also bear specific risks; otolaryngology and gastrointestinal procedures report elevated PRAEs due to airway manipulation and proximity to respiratory structures.^[6,8]^ Similarly, adenotonsillectomy performed for obstructive sleep apnea demonstrates high rates of postoperative respiratory challenges, requiring escalated admission to PICU for monitoring, in a retrospective cohort study,112 (63%) children after the surgery suffered AEs, of these, AEs occurred in 76 (42.5%) children in the PACU and in 106 (59.2%) of children in the PICU, 90% of all 76 children who suffered an AEs in the PACU had recurrent AEs in the PICU.^[9]^ Risk stratification based on surgical complexity and anticipated anesthesia duration is integral for optimizing perioperative and postoperative management plans.

### 4.3. Postoperative management variables

#### 4.3.1. Extubation timing and methods

Extubation decisions critically influence recovery outcomes and require careful evaluation of a child’s readiness for spontaneous breathing. Techniques such as awake extubation reduce the risk of airway collapse, eliminating residual neuromuscular blockade and ensuring optimal respiratory drive.^[23]^ Prolonged neonatal intensive care unit monitoring has highlighted the importance of timing in reducing reintubation risks, targeting those with low birth weight and postmenstrual age.^[7]^ Strategies addressing individual readiness and procedural coordination strongly mitigate RAEs risks associated with premature or delayed extubation practices. For premature or newborn babies, the appropriate volume-targeted ventilation for optimal weaning must be individualized, infants that remain ventilator dependent for extended periods because of increased anatomic dead space, under most circumstances, tidal volume should not be reduced below 4 to 5 mL/ kg during the weaning process to avoid excessive work of breathing. But regrettably, now it is still difficult to derive any recommendations concerning optimal weaning and extubation readiness in this vulnerable population at the moment.^[24]^

#### 4.3.2. PACU monitoring and interventions

PACU practices is essential for preventing and managing postoperative RAEs. Monitoring parameters such as oxygen saturation, airway patency, and respiratory activity provide early detection of emerging complications.^[13]^ Frequent assessments and interventions such as nasopharyngeal suctioning and respiratory support devices effectively reduce the incidence of hypoxemia and obstruction.^[20]^ Maintaining enhanced PACU surveillance for high-risk populations ensures timely intervention, reduces postoperative morbidity and prevents escalation to intensive care settings.^[25]^

#### 4.3.3. Impact of preoperative pulmonary testing

Preoperative pulmonary assessments significantly contribute to RAEs risk identification and stratification, and evaluation tools that measure parameters such as oxygen saturation nadir and polysomnography indices predict postoperative respiratory challenges, particularly in children with neuromuscular diseases or obstructive sleep apnea.^[17,26]^ By correlating the test results with surgical risks, clinicians can adopt targeted interventions to address weaknesses preoperatively. Incorporating polysomnographic findings into predictive modeling further enhances individualized risk management strategies.^[26,27]^

In synthesizing these factors-spanning patient characteristics, procedural nuances, and postoperative practices, a multifaceted approach is critical for minimizing RAEs in pediatric anesthesia recovery. The integration of personalized strategies informed by empirical evidence represents a decisive component of enhanced clinical outcomes and resource optimization.

## 5. Mechanistic insights

### 5.1. Pathophysiology and biomarkers

#### 5.1.1. Hypoxia-mediated inflammation and reactive airway responses

Hypoxia, defined as decreased oxygen availability at the tissue level, is a significant mechanistic factor in the development of RAEs during the postoperative recovery period in children receiving general anesthesia. Pediatric populations are particularly susceptible to hypoxia because of their unique airway characteristics and physiological immaturity. Hypoxia triggers a cascade of inflammatory responses in the respiratory system, mediated by the activation of hypoxia-inducible factors, which modulate cellular reactions to oxygen deprivation. These factors induce the release of pro-inflammatory cytokines, such as interleukin-6 (IL-6) and tumor necrosis factor-alpha (TNF-α), amplifying airway inflammation and increasing the likelihood of bronchospasm, laryngospasm, and other RAEs.^[25]^

Children with preexisting respiratory conditions, such as obstructive sleep apnea, asthma, or neuromuscular disease, show heightened vulnerability to RAEs due to chronic airway hypersensitivity and inflammation. For example, children with obstructive sleep apnea undergoing adenotonsillectomy are at an increased risk due to sustained inflammation from repetitive airway obstruction and subsequent hypoxia episodes during sleep. In these populations, postoperative observations have highlighted increased rates of desaturation, airway obstruction, and severe respiratory depression, necessitating rapid intervention.^[28]^

Additionally, nocturnal oxygen saturation nadir during polysomnographic assessments has been identified as a predictor of postoperative respiratory complications in children with preexisting conditions, such as neuromuscular disease. Reduced saturation underscores the risk of hypoxia-mediated inflammatory responses during recovery, reinforcing the need for careful preoperative evaluation and tailored intraoperative management.^[25]^ Moreover, individual risk factors such as anatomical predispositions and immature airway reflexes contribute to the severity of hypoxia-induced events.^[29]^

#### 5.1.2. Biological markers (e.g., red cell distribution width) as predictors

Biological markers play a crucial role in predicting RAEs, potentially enabling the early identification and proactive management of high-risk pediatric patients. One emerging biomarker in this context is red cell distribution width, a measure of erythrocyte size variability. Elevated red cell distribution width (RDW) has been linked to hypoxia-mediated systemic inflammation, making it an effective predictor of respiratory complications during recovery.^[17]^

A prospective study assessing RDW in children undergoing adenotonsillectomy for sleep-disordered breathing found that elevated preoperative RDW values were significantly correlated with increased postoperative respiratory events, including airway obstruction and oxygen desaturation. The study reported an adjusted odds ratio of 7.28 (95% CI: 4.30–13.28), emphasizing the predictive strength of RDW The cutoff value of 14.70 for RDW offered an area under the curve value of 0.74, supporting its clinical utility in preoperative planning.^[17]^

These findings suggest that RDW could be integrated into routine perioperative assessments, alongside polysomnographic indices, to enhance the risk stratification of pediatric patients undergoing anesthesia. In addition to RDW, other biomarkers such as eosinophil counts have been examined for their association with RAEs. A study observed increased incidence rates of cough, stridor, and hypoxemia in patients with eosinophilia during the perioperative period, demonstrating the importance of inflammatory markers in predicting adverse outcomes.^[30]^

Implementing biomarkers such as RDW and eosinophil counts, in clinical practice could streamline patient monitoring, enabling anesthesiologists to anticipate complications and tailor interventions accordingly. However, further studies are needed to validate the utility and scalability of incorporating multiple biological markers in pediatric anesthesia settings.

### 5.2. Implications of pediatric anatomic features

#### 5.2.1. Airway collapse and challenges related to structural immaturity

The anatomical differences between pediatric and adult airways significantly impact postoperative management during general anesthesia. Pediatric airways are characterized by smaller diameter, higher pliability, and larger relative tongue size than adults, which predispose children to airway collapse and obstruction.^[4,5]^ Furthermore, the immature structure of pediatric airways increases their susceptibility to compromised ventilation, especially during extubation or airway device removal.

These anatomical vulnerabilities are most pronounced in neonates and infants, whose cartilaginous structures are less rigid and prone to dynamic airway collapse under stress, such as suctioning or airway manipulation. Data from the APRICOT study demonstrated that critical respiratory events were disproportionately observed in younger children during airway interventions. Specifically, difficult airway management, including multiple device insertion attempts, results in higher incidence rates of RAEs, such as bronchospasm and laryngospasm.^[4]^

Age-related anatomical predispositions, combined with medical conditions such as sleep-disordered breathing, necessitate individualized airway management strategies. For example, tailored approaches to extubation, either under deep anesthesia or upon full awakening, must account for reduced structural stability and reactive airway tendencies in young children.^[5]^ These practices are critical to minimize the risk of collapse and ensure effective postoperative oxygenation.

#### 5.2.2. Interaction with neuromuscular agents and ventilation strategies

Neuromuscular blocking agents (NMBAs) are routinely used in pediatric anesthesia to facilitate surgical interventions and optimize airway management. Despite their utility, interactions between NMBAs and pediatric anatomical features can complicate the recovery process and exacerbate RAEs. In young patients, incomplete reversal of NMBAs is associated with an increased risk of postoperative pulmonary complications, such as atelectasis and prolonged hypoxemia, due to residual neuromuscular blockade impairing effective ventilation.^[31]^

Pressure-controlled ventilation remains the primary modality in pediatric anesthesia, as it offers improved lung protection and reduced risk of overdistension.^[31]^ In neonates and small infants, studies within the NECTARINE cohort indicated that consistent use of NMBAs with proper reversal, combined with pressure-controlled ventilation, was associated with lower incidence rates of postoperative pulmonary complications.^[31]^ These findings emphasize the importance of adopting appropriate ventilation strategies tailored to pediatric physiology to ensure both lung protection and effective gas exchange.

Postoperative management strategies should also consider the timing and degree of NMBA reversal prior to extubation. For example, failure to reverse NMBAs increases RAEs, whereas timely reversal mitigates such risks. Hence, the adoption of protocols that ensure complete NMBA reversal prior to extubation can significantly improve respiratory outcomes.^[31]^

Furthermore, the choice of anesthetic agents can influence respiratory outcomes. Propofol, with its reduced incidence of laryngospasm, may be preferred over agents such as sevoflurane in children undergoing procedures involving delicate airway management.^[32]^ This preference highlights the necessity of selecting anesthetic regimens and ventilation strategies that closely align with the anatomical and physiological peculiarities of pediatric patients to reduce perioperative complications.

## 6. Clinical recommendations

### 6.1. Preoperative strategies

#### 6.1.1. Early screening tools: STBUR questionnaire and pulmonary function testing

The identification of children at risk of RAEs begins with comprehensive preoperative screening. The STBUR questionnaire, designed to assess symptoms related to sleep-disordered breathing—such as snoring, trouble breathing, and feeling unrested, has demonstrated a significant association with perioperative respiratory complications. Screening positive for STBUR increases the odds of experiencing RAEs by 3.47 times, providing critical predictive value in clinical settings.^[33]^ Applying this tool can enable anesthesiologists to flag patients requiring specific perioperative measures, such as tailored anesthetic approaches or closer monitoring during the postoperative period.

Similarly, pulmonary function testing can aid in establishing the baseline respiratory status of pediatric patients, particularly those with chronic lung conditions or complex medical histories. Although there is some uncertainty about its routine application, pulmonary function testing offer valuable insights into the detection of underlying pulmonary dysfunction that could exacerbate postanesthesia complications.^[3]^ This evaluation is particularly important for children with neuromuscular or neurologic diseases, in whom diminished respiratory reserves may increase the risks during surgery.^[34]^

#### 6.1.2. Risk optimization through patient preparation and procedural delays

Preoperative optimization is pivotal for reducing RAEs in children with comorbidities or recent URIs. Studies indicate that URIs significantly increase the probability of perioperative respiratory complications, such as impaired oxygen saturation and laryngospasm.^[20,35]^ Delaying surgery has been shown to markedly reduce postoperative complications in children recovering from recent respiratory viral infections, particularly when the interval extends beyond 2 weeks.^[36]^ This strategy is critical for minimizing transient drops in oxygen saturation in the PACU and addressing fever or respiratory symptoms.

Preparatory measures may include noninvasive ventilation trials for high-risk patients, such as premature infants, newborn babies and personal history of respiratory system diseases, especially those with neuromuscular or chest wall diseases, to optimize oxygenation and minimize susceptibility to respiratory failure. Interventions such as bronchodilator therapy in children with asthma or airway hyperresponsiveness can bolster lung function preoperatively.^[11,27]^ These preparatory strategies underscore the value of recognizing modifiable risk factors and planning surgical timelines to improve the outcomes.

### 6.2. Intraoperative measures

#### 6.2.1. Selection of personalized anesthetic regimens

The choice of anesthetic technique is crucial for mitigating RAEs. Evidence from a randomized controlled trial revealed that intravenous induction with propofol results in a significantly lower incidence of perioperative RAEs than inhalational induction with sevoflurane, particularly in high-risk children.^[21]^ This aligns with meta-analytic findings confirming the advantage of propofol in reducing laryngospasm, vomiting, and agitation during recovery.^[32]^ However, the increased risk of apnea associated with propofol should inform careful procedural planning, including enhanced monitoring.

For special populations such as children on ketogenic diets, it is recommended to use intravenous agents with adjusted electrolytes and fluid management strategies to circumvent complications including metabolic acidosis or seizure exacerbation during postoperative recovery.^[37]^ Personalized anesthetic regimens should integrate specific patient characteristics, such as age, comorbidities, and anticipated procedural challenges, to optimize safety and effectiveness.

#### 6.2.2. Implementation of protective mechanical ventilation techniques

Protective mechanical ventilation strategies offer a pivotal opportunity to mitigate lung injury and to ensure adequate oxygenation during surgery. For pediatric patients, adherence to ventilation modalities such as pressure-controlled strategies is associated with reduced respiratory complications.^[31]^ Additionally, tailored tidal volumes and moderate positive end-expiratory pressure mitigate the risk of atelectasis and hypoxia, especially in neonates and infants whose immature physiology heightens their susceptibility.^[31]^

Children receiving NMBAs require careful reversal of the blockade before extubation, as failure to do so significantly amplifies postoperative pulmonary complications.^[31]^ Conversely, appropriate NMBAs management reduces critical respiratory events and enhances postoperative outcomes, particularly in neonates and small infants undergoing high-risk procedures.

### 6.3. Postoperative management guidelines

#### 6.3.1. Enhanced PACU surveillance protocols

Postoperative care protocols emphasizing enhanced monitoring in the PACU align with the need for the early recognition of respiratory complications. Key parameters included peripheral oxygen saturation levels, respiratory rate, secretion management, and detection of laryngospasm or bronchospasm during recovery. Studies confirm that children with preoperative URIs or those recovering from COVID-19 require comprehensive surveillance given their propensity for hypoxemia and airway obstruction.^[38,39]^ Enhanced vigilance, especially within the first few hours of recovery, ensures timely intervention, minimizes progression to critical events, and supports safer transitions to subsequent care settings.

Implementing structured monitoring systems is crucial for guiding PACU teams. For adolescent patients or those with prolonged anesthesia recovery times, staff should adopt frequent respiratory assessments to detect and address early signs of deterioration.^[40]^

#### 6.3.2. Use of advanced respiratory support devices in high-risk cases

The deployment of advanced respiratory support, such as continuous positive airway pressure and bilevel positive airway pressure is recommended for high-risk pediatric patients exhibiting compromised respiratory mechanics. Noninvasive ventilation techniques are particularly beneficial for children with neuromuscular disease or chronic lung conditions at risk of requiring reintubation.^[34]^ Additionally, extubation systematically transitioned to noninvasive ventilation and enhanced recovery trajectories by preventing lung collapse and maintaining oxygenation.^[34]^

Children with active respiratory symptoms, such as cough or fever, may benefit from adaptive respiratory interventions after post extubation. This differentiation in care ensures targeted support for vulnerable subsets within pediatric populations, ultimately improving postoperative respiratory stability. Advanced respiratory tools should be reserved for evidence-based scenarios in which utility can effectively mitigate severe postoperative complications effectively.^[41]^

These clinical recommendations detail evidence-based strategies to improve respiratory outcomes in pediatric patients undergoing general anesthesia. They emphasized the importance of preoperative identification and optimization, tailored intraoperative approaches, and vigilant postoperative care to mitigate RAEs comprehensively.

## 7. Future outlook and research directions

### 7.1. Development of predictive models

#### 7.1.1. Utilization of AI and multivariable modeling for RAEs forecast

Predictive models are essential for identifying at-risk pediatric populations prone to RAEs during postoperative recovery. The advent of artificial intelligence (AI) and machine learning methods has opened new possibilities in healthcare, especially in optimizing anesthesia practices. By analyzing extensive datasets, including patient demographics, surgical parameters, and anesthesia protocols, AI algorithms have the potential to deliver highly accurate forecasts of RAEs, offering critical insights into preventative action.^[16]^

Recent research has demonstrated how predictive analytics can be operationalized in clinical settings. For instance, predictive models derived from generalized estimating equations analyzing data from children with sleep-disordered breathing have successfully identified specific perioperative risk factors that contribute to RAEs, such as severe obstructive sleep apnea, prematurity, and preoperative oxygen use.^[16]^ These models validated the utility of algorithm-based approaches in processing multidimensional clinical data and extracting actionable risk patterns.

For AI applications to be fully realized, future research should focus on the creation of comprehensive and standardized datasets designed for training such models. Special focus should be given to incorporating variables, such as patient-specific anatomical predispositions, previous respiratory complications, and intraoperative management techniques. Moreover, ethical considerations regarding patient privacy must be addressed to ensure data security and compliance with regulatory standards. Longitudinally collected, multicentric data would further enhance the robustness and applicability of AI-driven insights across diverse pediatric populations.

The integration of predictive analytics into real-time clinical workflows also requires the development of user-friendly interfaces and decision support systems tailored to anesthesiologists. These tools could provide real-time feedback during perioperative monitoring with recommendations for optimizing extubation timing, mechanical ventilation strategies, or postanesthesia care based on predicted risks. Such methodologies would empower clinicians to anticipate complications and implement preemptive interventions tailored to individual patient profiles.^[16]^

#### 7.1.2. Validations of preoperative risk stratification systems

Preoperative risk stratification tools are indispensable to improve patient selection and procedural safety, Validated systems enable healthcare providers to categorize patients according to the likelihood of RAEs, thereby ensuring targeted interventions for vulnerable populations. Existing risk stratification frameworks, such as those developed for sleep-disordered breathing patients with features such as severe obstructive sleep apnea and airway hypersensitivity, have proven effective when applied to specific cohorts.^[16]^ However, these tools require broader validation across heterogeneous populations to improve their clinical utility.

Building on evidence-based findings, refinements to these stratification systems could include incorporating predictive markers identified in prior studies, such as prematurity and preoperative respiratory support requirements.^[16]^ Methodological approaches for validation must be centered on multicenter trials that encompass various surgical types, anesthesia protocols, and demographic-specific risk factors. Such studies would enable comparative assessments of stratification systems while identifying context-specific nuances that affect RAEs risks.

Furthermore, validated tools could play pivotal roles in multidisciplinary collaboration by providing patient counseling and procedural optimization efforts. For example, they can guide anesthesiologists in selecting less invasive airway management techniques or in adjusting preoperative medication protocols for specific at-risk profiles. Early screening measures and risk calculation could also direct institutional resource allocation, such as determining PACU monitoring intensity based on stratification scores.^[16]^ These implications underline the necessity of integrating validated tools within clinical pathways, transforming preoperative planning and anesthetic decision-making.

### 7.2. Expanding population studies

#### 7.2.1. Multicenter collaborative trials

Large-scale, multicenter collaborative trials represent a critical avenue for advancing the understanding of RAEs in pediatric anesthesia. By pooling resources across institutions, researchers can investigate disparities in the incidence and risk factors of RAEs across geographically and demographically diverse populations. Multicenter designs allow for the standardization of study protocols while providing representative data on RAEs associated with different surgical types, anesthesia techniques, and perioperative management strategies.^[16]^

These trials should aim to study the contextual influences on RAEs, such as regional variations in pediatric anesthesia practices, healthcare infrastructure, and patient health profiles. Additionally, exploring subgroup-specific outcomes would refine practitioners’ abilities to address differential risks. For instance, the factors influencing adverse events in ambulatory surgery settings may differ markedly from those in inpatient surgeries. Similarly, the effects of surgical duration and anesthesia type on RAEs warrant detailed investigation through collaborative research networks.

Collaborative efforts could also catalyze faster validation of predictive models and risk stratification systems by enabling comparative assessments across multiple cohorts. These initiatives can be complemented by partnerships with academic institutions and professional organizations, fostering data standardization, sharing methodologies, and maximizing the practical application of the resulting insights.^[16]^ Securing dedicated funding for multicenter studies from public health agencies or granting bodies is paramount to aligning objectives and facilitating meaningful outputs in this domain.

#### 7.2.2. Exploration into neonates, preterm infants, and special risk groups

Neonates, preterm infants, and other high-risk pediatric subgroups have unique vulnerabilities associated with physiological immaturity, making them particularly susceptible to RAEs. Despite being critical to the progress of anesthesia safety, research focusing on these demographics remains underdeveloped. The gaps in the literature relating to the specific anesthetic protocols and postoperative care requirements of such populations highlight the urgent need for targeted investigations.^[16]^

Special risk groups also encompass children with chronic respiratory conditions (like asthma) and congenital abnormalities impacting airway management. Tailored approaches to studying RAEs within these populations would provide critical data for planning preemptive measures. For example, clinical trials focusing on the effectiveness of therapeutic strategies such as preoperative pulmonary optimization or precision anesthetic regimens could mitigate predisposed risks.^[16]^

Collaborative research structures involving pediatric pulmonologists, neonatal intensivists, and anesthesiologists are essential for integrating domain-specialized expertise into study designs. Data derived from these populations could feed algorithmic models optimized for detecting both subtle and pronounced risk factors that predispose patients to RAEs.^[16]^ Additionally, refined knowledge from targeted studies would provide guidelines for adapting the equipment and monitoring protocols, such as those used in neonatal surgery.

A systematic focus on expanding population studies acknowledges the complexity of pediatric RAEs. Reliable insights generated from large-scale and specialty-focused trials can significantly enhance the safety and efficacy of pediatric anesthesia practices while optimizing care for the most vulnerable children.

## 8. Limitations

As a retrospective review, this study has the following 3 limitations: First, the data collection process did not include effect size meta-analysis, which precludes quantification of the overall population effect size. Future research should address this methodological gap. Second, the included study populations exhibited substantial heterogeneity, thereby limiting the generalizability of the findings. Third, the literature search was restricted to PubMed, Embase, and Cochrane Library databases without incorporating Chinese-language studies, potentially introducing selection bias. Consequently, the conclusions may not be applicable to certain populations. In subsequent studies, we will prioritize addressing these limitations.

## 9. Conclusion

### 9.1. Summary of core findings

#### 9.1.1. Major contributors to RAEs in pediatric anesthesia recovery

This review identified a comprehensive set of factors contributing to RAEs in pediatric patients recovering from general anesthesia. These factors included patient-related characteristics, procedural techniques, and postoperative management practices. Specifically, recent URIs, younger age (particularly neonates and infants), and preexisting conditions such as congenital abnormalities, sleep-disordered breathing, and asthma have been found to significantly increase the risk of RAEs.^[1,12,14]^

The procedural risk factors identified include the type of anesthesia induction (inhalational vs intravenous) and the choice of airway management strategy (e.g., cuffed or uncuffed endotracheal tubes). For instance, evidence strongly supports that intravenous induction with propofol can reduce the incidence of RAEs compared to inhalational induction with sevoflurane.^[21,22]^ Furthermore, surgeries involving anatomical structures close to the airway such as adenotonsillectomy are specifically associated with a higher prevalence of postoperative respiratory complications.^[9,15,17]^

Additionally, deficiencies in postoperative management, such as inappropriate timing of extubation, inadequate monitoring in the PACU, and insufficient supervision during transport, have been identified as significant triggers for RAEs.^[13,42]^ Weight thresholds below 18 kg and specific preoperative biomarkers such as elevated red cell distribution width, have also emerged as predictive factors for complications.^[15,17]^

#### 9.1.2. Their implications in improving efficiency and outcomes in perioperative care

Clinical understanding of these risk factors has profound implications for enhancing perioperative care efficiency. Recognizing that infants, children with recent URIs, or those with comorbidities are particularly vulnerable can refine preoperative screening protocols and inform decision-making about delaying surgery or adapting anesthesia strategies.^[1,19]^ Addressing RAEs can reduce postoperative complications and minimize PACU admissions and unplanned transfers to intensive care units.^[9,13]^

By making preoperative risk identification more thorough and equipped with tools such as polysomnography findings, red cell distribution width, or a stratified assessment of URIs symptoms, healthcare providers can focus on preventive measures that avert adverse outcomes.^[16]^ This not only shortens hospital stays but also efficiently utilizes healthcare resources, reduces costs and enhances recovery quality.^[13,15]^

### 9.2. Advocacy for improved pediatric anesthesia practices

#### 9.2.1. Focus on tailored anesthetic plans based on risk factors

Given the variability and unique physiology of pediatric patients, a move towards individualized anesthetic planning is imperative. This review advocates tailoring anesthetic regimens to accommodate the specific risk profiles of patients, particularly in high-risk groups such as those with obstructive sleep apnea, congenital heart disease, or recent respiratory infections.^[9,12,16]^ Intravenous induction techniques such as propofol should be considered a safer option for children at an elevated risk of RAEs.^[21,43]^

The advantages of personalized plans include optimized drug dosing, selection of airway management strategies, and adjustment of extubation processes to ensure safer emergence. For example, children with documented sleep-disordered breathing require extra vigilance during extubation and could benefit from preoperative bi-level positive airway pressure therapy.^[16]^

#### 9.2.2. Emphasis on continuous monitoring and quicker intervention strategies

Continuous monitoring in the immediate postoperative period remains critical, as many RAEs manifest symptoms during anesthesia emergence or shortly thereafter. Employing enhanced PACU surveillance protocols can significantly lower negative outcomes by enabling rapid responses to respiratory complications.^[9,13,20]^ Advanced monitoring devices, such as capnography and pulse oximetry with predictive algorithms can further aid in early detection.^[42]^

Training personnel in optimized pediatric airway management and equipping PACU with escalation mechanisms for intensive care can improve patient safety measures. For example, ensuring awake extubation over deep extubation in high-risk cases can reduce the likelihood of RAEs.^[29]^ Proactive measures in transport protocols, as emphasized in studies of anesthesia-related transport complications, would also mitigate the risks associated with transitioning pediatric patients to recovery or inpatient units.^[42]^

Advocacy for these practices underscores a commitment to addressing unmet needs in pediatric anesthesia care, ensuring safety, efficiency, and a holistic approach tailored to reduce perioperative complications. `

## Acknowledgments

The authors would like to thank all the reviewers who participated in the review.

## Author contributions

**Conceptualization:** Fang Li.

**Data curation:** Fang Li.

**Investigation:** Lihua Jiang.

**Methodology:** Yuhua Wu.

**Project administration:** Xiaoyan Zhou, Huan Yang.

**Software:** Anping Zhang.

**Writing – original draft:** Fang Li.

**Writing – review & editing:** Benzhen Chen.
